# A comparative study: the impact of different lipid extraction methods on current microalgal lipid research

**DOI:** 10.1186/1475-2859-13-14

**Published:** 2014-01-24

**Authors:** Yan Li, Forough Ghasemi Naghdi, Sourabh Garg, Tania Catalina Adarme-Vega, Kristofer J Thurecht, Wael Abdul Ghafor, Simon Tannock, Peer M Schenk

**Affiliations:** 1 School of Agriculture and Food Sciences, The University of Queensland, Brisbane QLD 4072, Australia; 2 School of Marine and Tropical Biology, James Cook University, Townsville City QLD 4811, Australia; 3 Australian Institute for Bioengineering and Nanotechnology and Centre for Advanced Imaging, The University of Queensland, Brisbane QLD 4072, Australia

**Keywords:** Microalgal oil, Fatty acid, Extract yield, Solvent polarity, Supercritical CO_2_, Lipid profile

## Abstract

Microalgae cells have the potential to rapidly accumulate lipids, such as triacylglycerides that contain fatty acids important for high value fatty acids (e.g., EPA and DHA) and/or biodiesel production. However, lipid extraction methods for microalgae cells are not well established, and there is currently no standard extraction method for the determination of the fatty acid content of microalgae. This has caused a few problems in microlagal biofuel research due to the bias derived from different extraction methods. Therefore, this study used several extraction methods for fatty acid analysis on marine microalga *Tetraselmis* sp. M8, aiming to assess the potential impact of different extractions on current microalgal lipid research. These methods included classical Bligh & Dyer lipid extraction, two other chemical extractions using different solvents and sonication, direct saponification and supercritical CO_2_ extraction. Soxhlet-based extraction was used to weigh out the importance of solvent polarity in the algal oil extraction. Coupled with GC/MS, a Thermogravimetric Analyser was used to improve the quantification of microalgal lipid extractions. Among these extractions, significant differences were observed in both, extract yield and fatty acid composition. The supercritical extraction technique stood out most for effective extraction of microalgal lipids, especially for long chain unsaturated fatty acids. The results highlight the necessity for comparative analyses of microalgae fatty acids and careful choice and validation of analytical methodology in microalgal lipid research.

## Background

Since the concept of using algae to make fuels was firstly discussed in the 1940s [1], a major focus for research, development and commercialization has become the cultivation of algae for the production of oil (lipid)-based products, in particular biodiesel through lipid transesterification. Algal lipids can be divided into two major types: polar lipids such as phospholipids and glycolipids, and neutral/non-polar lipids such as mono-, di- and tri-acylglycerides and carotenoids based on their physiochemical characteristics [2,3]. Some of these substances have been intensively studied, not only as biofuel feedstock, but also as beneficial food additives and other high-value products (e.g., eicosapentaenoic acid (C20:5 n-3, EPA), docosahexaenoic acid (C22:6 n-3, DHA) and other long-chain polyunsaturated fatty acids (LC-PUFA)) [4,5]. Therefore, there is mounting interest on investigation of microalgal potential for production of food commodities and fatty acids bound as triglycerides for nutraceutical efficacy in recent decades [6]. Significant advances have been made in upstream processing to generate cellular biomass for lipid yields. However, as part of the downstream process, lipid extraction continues to be a significant challenge towards the commercial production of microalgal oil production, even though a multitude of extraction methods have been described in the literatures.

For microalgal oil extraction, although an appropriate technique of cell disruption is a prerequisite [7,8], the efficient extraction of lipids is highly dependent on the polarity of the organic solvent or solvent mixture used [9,10]. In general, solvent mixtures containing a polar and a non-polar solvent could extract a greater amount of lipids [11]. For example, a combination of chloroform (non-polar), methanol (polar) and water, known as the Bligh & Dyer method, has been used for lipid extraction from a wide range of biological samples [11]. However, concerns about biosafety issues using extraction solvents has driven a demand for biocompatible and less or non-toxic solvents (e.g., dichloromethane) [12]. Alternative solvent methods for lipid extraction thereby have been studied; for instance, saponification has resulted in significant lipid recoveries from several types of microalgae [7,13-16]. In recent years, supercritical fluid technology has been adopted for microalgal oil extraction, especially for pharmaceutical and neutraceutical bioproducts. In comparison with liquid solvent extractions, the supercritical fluid carbon dioxide (ScCO_2_) technique offers several advantages, such as no toxicity, no oxidation or thermal degradation of extracts, high diffusivity and easy separation of desired bioproducts [16-18]. However, it has been reported that lipid yield using ScCO_2_ extraction was much lower than employing the Bligh & Dyer method on heterotrophically cultured microalgae of *Crypthecodinium cohnii*[19]. At present, comparative economics of technical and physiochemical methods for oil extraction have not been accomplished on microalgae cells.

Given the large diversity of microalgae species, the ability to successfully and effectively extract oil from cellular biomass becomes paramount in determining the yield and suitability across oleaginous strains [18,20]. However, the current research attention towards oil extraction from microalgae has been predominantly focused on the potential energy efficiency and cost effectiveness of the methods themselves. Despite the differences in extraction efficiency obtained depending on different extraction methods [10-12,21,22], there is little attention on the bias potentially derived from different extraction methods, in particular when screening optimal microalgal species for lipid-based bioproducts. Due to the lack of a standard extraction method for fatty acids (FA) analysis, therefore, the motivation behind this study was to investigate the potential impact of different lipid extraction methods on microalgal lipid research.

The present work includes a comparative study of lipid extractions from lyophilised biomass of the oleaginous green alga *Tetraselmis* sp. Soxhlet extraction was conducted for lipid recovery using either single solvents or mixtures. In addition, algal biomass was used for five different extraction methods that were successfully used for efficient algal lipid extraction in previous studies. These parallel extraction methods were: (1) the monophasic ternary system of chloroform:methanol:water, one of the most commonly used methods for lipid extractions [23]; (2) a less hazardous solvent mixture of dichloromethane:methanol [12]; (3) another alternative solvent mixture of propan-2-ol:cyclohexane:water recommended by Schlechtriem et al. [24]; (4) direct saponification using KOH in ethanol [7] and (5) supercritical CO_2_ extraction [25]. We discuss and draw some parallels with these extractions to highlight the differences on extractable lipid production and hydrolysed fatty acid methyl ester profiles on microalgae cells.

## Results and discussion

### The impact of solvent polarity on lipid extraction

**Figure 1:**
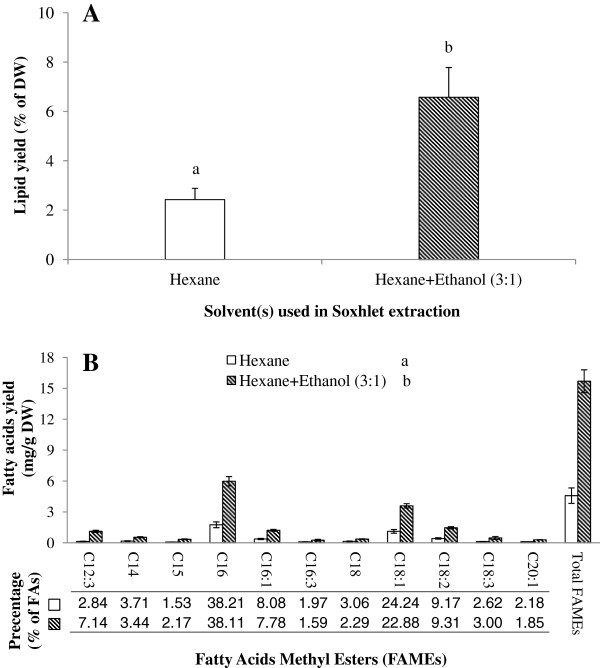
**Comparison of lipid recovery by Soxhlet extraction utilising hexane and hexane-ethanol (3:1) for A) Lipid yields and B) FAMEs profile.** Different letters represent a significant difference between hexane and hexane-ethanol (*P* < 0.05).

The results obtained for Soxhlet extraction of microalgal lipids showed a significant difference in extraction efficiency between hexane and the mixture of hexane and ethanol in both, total lipids and total FAMEs, as well as each individual fatty acid (*P* < 0.05, Figure 1A and B). As ethanol is a polar solvent, it can extract more polar lipids and likely penetrate the cell wall, hence making triacylglycerides (TAGs; neutral lipids) more available for the non-polar solvent hexane. The lipid extraction yield in the mixture was nearly three times higher than when using hexane alone (Figure 1A). Coincident with the reports of Ryckebosch et al. [11] and Lewis et al. [10], it seems that extraction solvents containing a mixture of a polar and a nonpolar solvent could extract higher amounts of lipids and also some other compounds (e.g., pigments, carbohydrates and algaenans) [26]. Interestingly, this conclusion contradicts the study of Shen et al. [27] stating that 1:1 (v/v) of hexane and ethanol had less lipid yields than hexane on *Scenedesmus dimorphus* and *Chlorella protothecoides*. Regardless of the biological difference of these algal species and its resulting different lipid class compositions, the contradiction is possibly also related to the different proportions of hexane:ethanol in the mixture (3:1 vs. 1:1). A similar result was obtained using other mixtures as well, such as chloroform-methanol [11] and hexane-hydroalcoholic solution [28] where different ratios of solvents also resulted in the different extraction efficiencies on microalgal lipid extraction. Therefore, it is implied that only appropriate proportions of polar and nonpolar solvents could achieve higher yields of lipid compared with single solvent extraction.

Although the Soxhlet extraction method has been used for a range of biological organisms [18,29,30], Soxhlet extraction is extremely time-consuming [22,30]. It also could cause thermo-degradation of LC-PUFAs (e.g., ω-3 fatty acids) [29]. Although the efficiency of Soxhlet extraction could be improved significantly by using solvent mixtures, the extraction yields (percentage of extracts in algal dry weight) were still lower than the values obtained in the parallel extractions (Table 1). The inefficiency of Soxhlet extraction has also been reported in other studies [e.g.,[3,22]. Therefore, the Soxhlet extraction method was excluded in the lipid extraction comparison in this study.

**Table 1:** Comparison of extract content between different lipid extraction methods

	**Chl:Met**	**Dic:Met**	**Pro:Hex**	**Eth:KOH**	**ScCO**_**2**_
**Extract content (% of dry weight)**	11.66 ± 1.16 (abc)	15.05 ± 0.46 (a)	13.35 ± 1.15 (ab)	9.40 ± 1.64 (c)	10.88 ± 0.46 (bc)

Different small letters indicate significant differences using one-way ANOVA analysis (*P* < 0.05).

Chl:Met: chloroform and methanol method;

Dic:Met: dichloromethane and methanol method;

Pro:Hex: propan-2-ol and cyclohexane method;

Eth:KOH: ethanol and KOH method;

ScCO_2_: supercritical-CO_2_ extraction method.

### Determination of lipid content in the microalgal dry biomass

The Thermogravity Analyser (TGA) measures the change of weight of various materials at given temperatures while the temperature is increased over time [31]. Through the comparison between initial and defatted biomasses, the temperature range of TGA selected in this study was correlated to the lipid content in microalgae. Meanwhile, it is worth noting that there was still a bit of moisture content in the lyophilised biomass within 25-190°C, showing the difference before and after lipid extraction. Therefore, normalised microalgal biomass (via water deduction) is more appropriate for lipid quantification, which is different from conventional gravity measurements. As the accuracy in TGA analysis of algal biomass can reach microgram levels, the application of TGA will be a useful analytical framework for assessing lipid yields from microalgae, especially for microalgal biodiesel research [26].

Our data show that the production of lipid extracts was significantly different among the five extraction methods tested (*P* = 0.029, Table 1). The mean value of lipid content was between 9.4% and 15.05% in lyophilised *Tetraselmis* sp. M8 biomass. The yield obtained from the mixture of dichloromethane and methanol (Dic:Met), was much higher than those from direct saponification (Eth:KOH) and supercritical-CO_2_ extractions (ScCO_2,_*P* < 0.05). The extraction yield from the propan-2-ol and cyclohexane method (Pro:Hex) was also significantly higher than that from Eth:KOH (*P* < 0.05). The extraction yield from the Bligh & Dyer method (Chl:Met) was not statistically different to yields from any of the other methods used (*P* > 0.05). In terms of lipid yields, the order of extraction efficiency on *Tetraselmis* sp. M8 could be ranked as Dic:Met > Pro:Hex > Chl:Met > ScCO_2_ > Eth:KOH. However, this sequence was not applicable for other microalgae. For example, a contradicting result was observed on the microalga *Crypthecodinium cohnii* that the lipid yield attained from Chl:Met was nearly double that of ScCO_2_[19]. Although it is likely associated with a different extraction process, the effectiveness of a lipid extraction method may also be dependent on the microalgal species used [8,10,22]. Differences can be explained by differences in size and in particular cell wall composition. Therefore, a comparative analysis of microalgal fatty acids and choice and validation of analytical methodology are essential for microalgal lipid research.

Interestingly, the sequence of gravimetrically-measured lipid yields was not equivalent to the order of the FA content when quantifying FAME by GC/MS (Table 2). The total fatty acid content determined by GC/MS varied between 6 to 10% of dry weight (DW). In our comparison, the maximum yield of total FA was achieved through ScCO_2_ (10%), followed by Dic:Met (8.64%), Chl:Met (8.33%), Pro:Hex (8.18%) and Eth:KOH (6.06%). Discrepancies between both methods ranged from 0.88% for ScCO_2_ to 6.41% for Dic:Met (Table 1, 2). Similar to this study, such a difference was also observed in the oil extract on *Botryococcus braunii*[32], because of the co-extraction of other compounds (e.g., non-polysaccharide biopolymers, polyaldehydes and polyacetals [33]). Although a further investigation will be needed to identify and quantify these components in *Tetraselmis* sp. M8 biomass, it is also in some ways surprising given the many years dedicated by others to elucidating both lipids and the other chemical compounds as “oil/lipid” content in microalgae [26]. Clearly, the amount of co-extracted non-TAGs in the “lipid” fraction varies for different extraction methods and algal strains, making a comparison of lipid yields across different laboratories and microalgal species extremely difficult. Therefore, only the content of FAME identified by GC/MS, was considered as a useful measure to assess lipid production in this study.

**Table 2:** Comparison of normalised fatty acids (FA) composition between different extraction methods (% of dry weight) determined from FAME analysis by GC/MS

	**MW**	**Chl:Met**	**Dic:Met**	**Pro:Hex**	**Eth:KOH**	**ScCO**_**2**_
***C14***	242	0.03 ± 0.01 (a)	0.06 ± 0.01 (ab)	0.12 ± 0.03 (b)	0.06 ± 0.02 (ab)	0.19 ± 0.01 (c)
C16	270	3.57 ± 0.14	3.63 ± 0.37	3.27 ± 0.44	3.31 ± 0.32	3.81 ± 0.47
***C16:1 (n-7)***	268	0.14 ± 0.08 (a)	0.06 ± 0.01 (a)	0.09 ± 0.02 (a)	-- (b)	0.09 ± 0.03 (a)
***C16:1***	268	0.39 ± 0.03 (a)	0.59 ± 0.05 (ab)	0.58 ± 0.12 (ab)	0.37 ± 0.06 (a)	0.81 ± 0.08 (b)
***C16:2***	266	0.06 ± 0.02 (a)	0.07 ± 0.01 (a)	0.12 ± 0.03 (a)	-- (b)	0.21 ± 0.00 (c)
C16:3	264	0.44 ± 0.05	0.36 ± 0.06	0.38 ± 0.11	0.35 ± 0.05	0.31 ± 0.02
***C18***	298	0.44 ± 0.04 (a)	0.27 ± 0.01 (b)	0.43 ± 0.05 (a)	0.34 ± 0.02 (ab)	0.38 ± 0.02 (ab)
C18:1 *(n-9c)*	296	0.98 ± 0.47	1.26 ± 0.13	1.17 ± 0.08	0.89 ± 0.07	1.25 ± 0.53
***C18:1 (n-9t)***	296	-- (a)	0.06 ± 0.03 (a)	0.06 ± 0.01 (a)	-- (a)	0.16 ± 0.02 (b)
C18:2 *(n-6)*	294	0.72 ± 0.07	0.52 ± 0.12	0.62 ± 0.07	0.68 ± 0.13	0.73 ± 0.18
***C18:3 (n-6)***	292	0.15 ± 0.03 (a)	0.16 ± 0.01 (a)	0.04 ± 0.01 (b)	0.05 ± 0.01 (b)	0.12 ± 0.03 (a)
***C18:3 (n-3)***	292	0.25 ± 0.02 (ab)	0.43 ± 0.04 (c)	0.47 ± 0.05 (c)	0.24 ± 0.07 (a)	0.41 ± 0.05 (bc)
C20	326	0.03 ± 0.02	--	--	0.06 ± 0.04	0.04 ± 0.05
***C20:1 (n-9)***	324	0.50 ± 0.06 (ab)	0.36 ± 0.03 (a)	0.47 ± 0.04 (ab)	0.37 ± 0.02 (a)	0.60 ± 0.09 (b)
***C20:4 (n-6)***	318	0.12 ± 0.00 (a)	0.20 ± 0.03 (b)	0.06 ± 0.02 (a)	0.12 ± 0.02 (a)	0.27 ± 0.03 (b)
***C20:5 (n-3)***	316	0.47 ± 0.08 (ab)	0.32 ± 0.04 (abc)	0.27 ± 0.05 (bc)	0.21 ± 0.07 (c)	0.52 ± 0.06 (a)
***C22:5 (n-3)***	344	-- (a)	-- (a)	-- (a)	-- (a)	0.03 ± 0.01 (b)
***C22:6 (n-3)***	342	-- (a)	-- (a)	0.04 ± 0.00 (b)	-- (a)	0.04 ± 0.00 (b)
Total Saturated FA *(mean% of total FA)*		48.65	47.43	46.98	46.83	44.36
Total Monounsaturated *(mean% of total FA)*		25.21	27.76	28.66	27.01	29.17
Total Polyunsaturated *(mean% of total FA)*		26.14	24.81	24.36	26.16	26.47
***Total FA (mean% of dry weight)***		8.33 ± 0.30 (a)	8.64 ± 0.49 (ab)	8.18 ± 0.51 (a)	6.06 ± 0.44 (c)	10.00 ± 0.27 (b)

Chl:Met -- chloroform and methanol method; Dic:Met -- dichloromethane and methanol method; Pro:Hex -- propan-2-ol and cyclohexane method; Eth:KOH -- ethanol and KOH method; ScCO_2_ -- supercritical-CO_2_ extraction method.

Notes: Values less than 0.03% were deleted from the calculation to eliminate the effect of background and labelled as “--“ in the table. The Bold and Italic parameters in the first column were dependent on extraction method, indicated by different small letters in brackets which show significant differences between extractions methods (*P* < 0.05).

### The impact of different extraction methods on microalgal fatty acids yield

**Figure 2:**
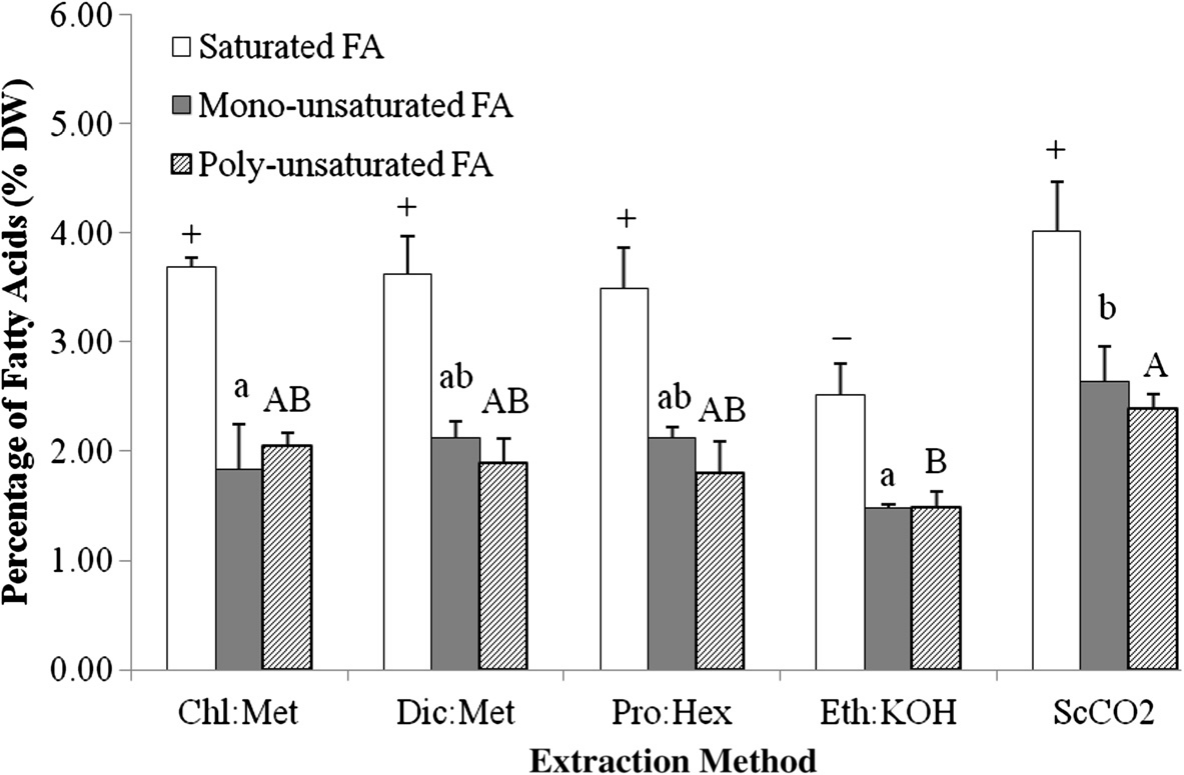
**Total amount of saturated, mono- and polysaturated fatty acids in microalgal dry biomass (%) across different extraction methods.** Chl:Met -- chloroform and methanol method; Dic:Met -- dichloromethane and methanol method; Pro:Hex -- propan-2-ol and cyclohexane method; Eth:KOH -- ethanol and KOH method; ScCO_2_ -- supercritical-CO_2_ extraction method. Different symbols, small and capital letters represent significant differences on saturated, mono- and polysaturated fatty acids, respectively, for the different extraction methods (*P* < 0.05).

The saturated fatty acids (SFA), monounsaturated fatty acids (MUFA) and PUFA were all obtained in five extraction methods from *Tetraselmis* sp. M8 biomass, but with different yields (*P* < 0.05, Figure 2). A significant difference was only observed between ScCO_2_ and Eth:KOH, where the FA yields were much lower in the latter (*P* < 0.05, Figure 2). Given the lower FA yields, Eth:KOH clearly shows a lack of competence for FA extraction. However, this is could be specific to *Tetraselmis* sp. M8, since direct saponification was quite successful for the lipid yield and better than liquid solvent extractions for other microalga species, such as *Thraustochytrium* sp. [7], *Isochrysis galbana*[21] and *Phaeodactylum tricornutum*[14]. On the other hand, it demonstrates the importance of testing different extraction methods for different microalgae.

Although the results showed no statistically significant difference between Dic:Met, Pro:Hex and ScCO_2_ (*P* > 0.05), the mean values of FA yields were relatively higher for ScCO_2_. Additionally, the yield from ScCO_2_ can be significantly increased by using wet algal -paste rather than dry biomass [34]. This is because supercritical CO_2_ is a non-polar solvent and the water will act as a natural polar co-solvent [22]. As the biomass used in this study was lyophilised, further improvements for ScCO_2_-based lipid extraction may be achieved through the presence of water that can facilitate polar extractions. It is conceived that ScCO_2_ is more efficient to extract more FA yield than other methods. More importantly, energy consumed in the drying process can be reduced by using supercritical extraction technology [22], which would be important from a commercial perspective.

Generally yields between Chl:Met, Dic:Met, Pro:Hex and Eth:KOH (excl. the lower amount of SFA in Eth:KOH, Figure 2) did not differ widely. With concerns about the safety and hassles of using chloroform for microalgal biofuel research, this study suggests that dichloromethane could readily replace chloroform for microalgal lipid extraction. This conclusion is also applicable to plant and animal materials for which Dic:Met and the Bligh & Dyer method (Chl:Met) also gained similar FA yields [12]. For *Tetraselmis* sp. M8 lipid extraction, the non-chlorinated solvents, propan-2-ol and cyclohexane present another alternative to the Chl:Met method. This result is consistent with Chl:Met and Pro:Hex lipid extraction data on *Ditylum brightwellii*[24].

### The difference of fatty acid methyl esters (FAME) among five extraction methods

Overall, our comparison highlights that different extraction methods not only could lead to different FA yields (Figure 2), but also affect the FA profile to a large extent (Table 2). Only a few fatty acids were not significantly influenced (*P* > 0.05). However, it is worth noting that they were the most abundant FAs in the lipid extracts (approx. 70% of total FAs), such as C16 hexadecanoic (or palmitic) acid, C18:1 (n-9) oleic acid and C18:2 (n-6) octadecadienoic (or linoleic) acid. These FAs are normally treated as the major components for microalgal biodiesel production [34]. Despite of differences in total lipid yield, it is conceived that these different extraction methods maybe less relevant for microalgal biodiesel research, demonstrated by the similar amount of these dominant FAs.

However, the extraction effectiveness on most long chain unsaturated FAs, was significantly dependent on the extraction method (*P* < 0.05, Table 2). When using ScCO_2_ for extraction, the yield for each FA was also almost ranked highest. The basic principal of this technology is achieving a certain phase (supercritical) that is beyond the critical point of a fluid, in which the meniscus separating the liquid and vapour phases disappears, leaving only a single homogeneous phase [35]. Consequently, the changes of the thermophysical properties transform the fluid into a super-solvent and thus, could improve extraction and reaction efficiency [34]. Moreover, ScCO_2_ likely shows a better performance on unsaturated FA extraction, demonstrated by its relatively lower proportion of SFA (44.36% of total FA). This is coincident with previous reports that there is a low risk for lipid oxidation or thermal degradation during ScCO_2_ extraction [16-18]. With this regard, ScCO_2_ extraction would be more meaningful for high value FAs studies in microalgae.

**Figure 3:**
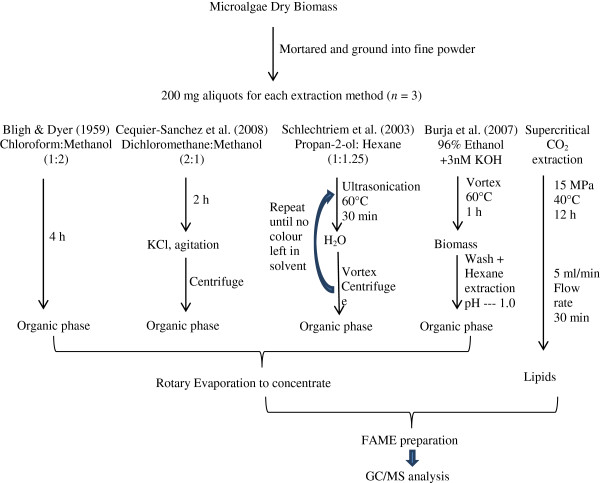
Brief overview of lipid extraction methods used.

Similar to the ScCO_2_ extraction, a small amount of DHA was also observed in the Pro:Hex method (Table 2). It cannot be ruled out that this stems from the contribution of both thermal bath and ultrasonication treatments during the Pro:Hex extraction (Figure 3). As a benchmark commonly used for lipid extraction, the Bligh & Dyer method (Chl:Met) was not successful with for extraction of C22:5 (n-3) and C22:6 (n-3) (DHA). This was also not doable when using Dic:Met or saponification (Eth:KOH). Furthermore, the amount of other long chain FAs (e.g., C18:3 (n-3), C20:4 (n-6) and C20:5 (n-3) which are linoleic acid (ALA), eicosatetraenoic acid (ETA) and eicosapentaenoic acid (EPA)) was also significantly different for these extractions (*P* < 0.05, Table 2). At this point, the feasibility of Dic:Met and/or Pro:Hex as an alternative for Chl:Met as suggested above, would be worth considering for lipid profile analyses in microalgae.

## Conclusion

Through comparison of extraction methods, this study highlights the bias on microalgal lipid recovery, demonstrated by clear differences in microalgal lipid production and FAME profile analyses. As a consequence, different lipid extraction methods selected for microalgal lipid studies can result in widely varying estimations of the lipid-based bioproducts of microalgae. As outlined in previous studies [4,26], the lipid profile and production yields are also highly dependent on microalgal cultivation conditions, biomass processing, cell disruption and strain selection in addition to solvent polarity and extraction processing. In such a scenario, meaningful data for microalgae under consideration for high value products and biodiesel production will require careful choice and validation of analysis methodology. In the present comparison, this study would highly recommend the supercritical CO_2_ technique for lipid extraction, aiming for an accurate evaluation on the potential of microalgae for high value FA production. Meanwhile, this study also can serve as model for how such studies would be conducted across algal genera that produce triglycerides as their main biodiesel feedstock. From a commercial perspective, a techno-economic assessment is needed and should ideally be carried out for large-scale extraction where costs are likely to be very different compared to the presents laboratory-based study.

## Methods

### Experimental microalgae

Marine microalga *Tetraselmis* sp. (strain M8) was isolated from the Sunshine Coast, Queensland, Australia (26°39′39″S, 153°6′18″E; Genbank accession number JQ423158). By using a 2 × 1,000 L split microalgal cultivation system M8 culture was scaled up and induced for lipid accumulation by nutrient deprivation as described previously [36]. The freeze-dried biomass was ground into a fine powder for subsequent extractions.

In order to improve our understanding and to highlight the importance of extraction method selection, microalgal oil extraction was conducted with two *modi operandi*. First, Soxhlet extraction was performed with either single solvents or a mixture of solvents, and conventional gravimetric methods along with fatty acids analysis through GC-MS were used for quantification and qualification of lipid extraction. The other approach entailed comparative extractions by five different methods, coupled with a technique of Thermo Gravity Analysis (TGA) for microalgal lipid content determination. All solvents used for lipid extractions were HPLC grade.

### Soxhlet extraction: single solvent *vs.* mixture

The Soxhlet extraction was implemented with 2 g of lyophilised *Tetraselmis* sp. M8 biomass powder on a Soxhtec system HT (Foss Soxtec 1043): 6 hours of extraction process at 140°C, followed by 30 min solvent rinse and 30 min solvent evaporation. There were two extraction solvent schemes for lipid recovery: 52 ml hexane alone and the mixture (39 ml hexane + 13 ml ethanol) (*n* = 3). The weight of oily extract was weighed and counted as oil content (% DW) and subsequent fatty acid analyses were carried out by GC-MS.

### Comparison of five lipid extractions

Comparative lipid extractions were carried out with 200 mg aliquots of microalgal powder by five different approaches (*n* = 3, Figure 3). The first extraction method was following Bligh & Dyer [23] with minor modifications. Briefly, the algal powder was eluted by 5 ml of chloroform and methanol (1:2, v/v; CHCI_3_/MeOH) in a capped glass tube, and placed in an Ultrasonic Cleaner (Unisonics N1984) at room temperature. With an interval of one hour, the samples were added with 2 ml CHCI_3_ and 3.6 ml water, vigorously vortexed and centrifuged at 1,000 × g for 5 min. The organic phase was pipetted into a new glass tube, and replaced by the same amount of CHCI_3_ to maintain the extraction volume in the extraction tube for re-extraction. About 4 hours later (4 rinses) when there was no colour appearing in the freshly-added solvent, all organic layers were pooled together and then evaporated using a rotary evaporator (Buchi Rotavapor RE120).

The second extraction method was adopted from Cequier-Sanchez et al. [12]. First, 200 mg of the dry biomass was extracted by immersion in 6–8 ml of dichloromethane-methanol (2:1, v/v; CH_2_CI_2_/MeOH) contained in a capped glass test tube, performing occasional gentle hand agitation for 2 hours. Subsequently, the samples were filtered through a glass fibre filter paper under vacuum and transferred to a new test tube. A total of 1.25 ml of KCI aqueous solution (0.88%, w/v) was added into the filtration, followed by strong agitation and centrifugation at 1,500 × g at 4°C for 5 min. The aqueous phase was discarded, whilst the organic phase was collected for rotary evaporation.

The third method was using propan-2-ol and cyclohexane (1:1.25, v/v; C_3_H_8_O/C_6_H_12_) as described by Schlechtriem et al. [24]. The samples were put into the test tubes and mixed with 9 ml of C_3_H_8_O/C_6_H_12_, followed by 30 s vortexing. Then, the tubes were ultrasonicated at 60°C for 30 min (Unisonics Australia). Then, 5.5 ml of water was added to obtain a mixture with C_3_H_8_O/C_6_H_12_. After 30 s of vortexing, the different phases were separated by centrifugation at 1800 × g for 10 min. When the organic phase was transferred to a new test tube, the sample was extracted again with adding 5 ml of C_3_H_8_O/C_6_H_12_. Such a repeated extraction was ceased after the fifth time when the extract colour became invisible in the organic phase. Similar to the first extraction method, all the organic phases were pooled together and evaporate-concentrated.

The fourth extraction was conducted by direct saponification, adopted from Burja et al. [7]. Briefly, the samples were immersed in 15.2 ml of 3 mM KOH in 96% ethanol in the test tubes. Then the tubes were vortexed at 60°C for 60 min. Samples were cooled to room temperature and filtered as above. The biomass was washed with 4 ml of ethanol and all the alcoholic solutions (incl. the first filtration) were transferred to a graduated mixing cylinder, and 4 ml of water was added. The unsaponifiables were further extracted by adding 8 ml hexane and gently shaking twice. When the layers were separated, the pH was decreased to 1 by adding HCI/H_2_O (1:1, v/v) solution. Then, both saponifiable and unsaponifiable lipids in the top layer were recovered by two rounds of addition of 4 ml hexane and gentle mixing. Then the organic layer was evaporated.

Supercritical-CO_2_ extraction of microalgal lipids was performed with commercial-grade CO_2_ in the supercritical facility within the Australian Institute for Bioengineering and Nanotechnology (AIBN) at The University of Queensland. The algal samples were placed in a small glass tube located in a 60 ml extractor. Typically, extraction was carried out with an initial soaking period of 12 h (15 MPa at 40°C). This was followed by a flushing cycle in which CO_2_ was flowed over the sample at a flow rate of 5 ml/min controlled by an ISCO syringe-pump for 30 min.

All the extracts from above were collected and preserved at 4°C for lipid profiling analysis as below. As microalgae possess a large amount of natural antioxidants, addition of antioxidants was not needed for lipid extraction when short expression times were used [11].

### Quantification of the extract content in microalgal dry biomass

Posterior to the five parallel lipid extractions, the algal residue (defatted biomass) was collected and lyophilised again (10 h), then analysed on a Thermogravimetric Analyser (TGA/DSC 1 *Star e System*) (*n* = 3). The setting was with nitrogen at a flow rate of 50 ml min^-1^, at a programmed heating interval of 10°C min^-1^, until reaching 550°C. According to our preliminary study, the algal biomass reduction that occurred between 190 and 540°C represented the major difference between algal cells and extracted algal cells as demonstrated using the *Tetraselmis* sp. M8 sample. The range 190-540°C was therefore selected as the effective temperature range for the extracts in the biomass. The mass loss of water residue in the algal biomass (25-190°C) was then deduced to normalise the microalgal biomass loss in the TGA analysis. The difference between original algal biomass and defatted sample indicated the amount of materials being extracted, based on the formula:

$$\mathrm{Extract}\;\mathrm{content}\left(\%\right)=\frac{\mathrm{Biomass}\;\mathrm{reduction}\left(190-540{}^{\circ}C,\mathrm{mg}\right)}{\mathrm{Normalised}\;\mathrm{biomass}\;\mathrm{loss}\left(\mathrm{mg}\right)}\times 100$$

### Fatty acid methyl ester (FAME) analyses

The condensed lipid extracts were hydrolysed and methyl-esterified for FAME analysis by GC-MS [36]. Briefly, 100 *μ*l of extract were mixed with 500 *μ*l of 2% H_2_SO_4_/methanol solution in a 2 ml eppendorf tube by shaking at 80°C for 2 h. In each sample, 100 *μ*g of heneicosanoic acid (Sigma, USA) was added as an internal standard prior to the reaction. A total of 500 *μ*l of 0.9% (w/v) NaCl and 500 *μ*l of hexane was then added to the sample which was subsequently vortexed for 20 s and centrifuged at 16,000 × g for 3 min. The hexane layer was pipetted into an autosampler vial for FAME quantification. 1 *μ*l of the hexane layer was injected into an Agilent 6890 gas chromatograph equipped with a 5975 MSD mass spectrometer (Agilent Technologies Australia Pty Ltd; GC/MS), for identification of FAMEs. Separation was achieved on a DB-Wax column (Application note: 5988-5871EN) with a cyanopropyl stationary phase with helium as carrier gas in constant pressure mode. Identification of FAME was based on mass spectral profiles, comparison to standards, and expected retention time from Agilent’s RTL DB-Wax method (Application note: 5988-5871EN). In the end, all FAME data were normalised in percentage of dry weight to allow the comparative analysis between different extractions.

### Data analysis

The variation of FAs and lipid contents between extraction methods was investigated by one-way ANOVA, with a Least Significant Differences (LSD) procedure for the post hoc comparisons. A significance level of *P* < 0.05 was used for all tests.

## Competing interests

The authors declare no competing interests.

## Authors’ contributions

YL and FG contributed to the experimental design, data acquisition, troubleshooting, analysis and interpretation of data, as well as drafting the manuscript. KJT and WAG helped in sample preparation and execution of work on TGA and ScCO_2_. All authors contributed in data collection from literature and writing of the manuscript. All authors have read and approved the final manuscript.
